# Rapid virucidal activity of an air sanitizer against aerosolized MS2 and Phi6 phage surrogates for non-enveloped and enveloped vertebrate viruses, including SARS-CoV-2

**DOI:** 10.1128/aem.01426-24

**Published:** 2024-12-06

**Authors:** M. Khalid Ijaz, Bahram Zargar, Raymond W. Nims, Julie McKinney, Syed A. Sattar

**Affiliations:** 1 Global Research & Development for Lysol and Dettol, Reckitt Benckiser 544446 , Montvale, New Jersey, USA; 2 CREM Co. Labs., Mississauga, Ontario, Canada; 3 Syner-G BioPharma, Boulder, Colorado, USA; 4 Faculty of Medicine, University of Ottawa 6363 , Ottawa, Ontario, Canada; Centers for Disease Control and Prevention, Atlanta, Georgia, USA

**Keywords:** aerobiology chamber, airborne virus, air sanitization, phage MS2, dipropylene glycol, phage Phi6, viral inactivation

## Abstract

An air sanitizer was evaluated using an aerobiology protocol, compliant with the U.S. Environmental Protection Agency’s Air Sanitizer Guidelines, for virucidal activity against bacteriophages Phi6 and MS2 (used as surrogates for enveloped and non-enveloped human pathogenic viruses). The phages were suspended in a medium containing a tripartite soil load simulating body fluids and aerosolized using a six-jet Collison nebulizer in an enclosed 25 m^3^ aerobiology chamber at 22 ± 2°C and 50 ± 10% relative humidity. The air sanitizer was sprayed into the chamber for 30 s. Viable phages in the air were captured directly, in real time, on host bacterial lawns using a slit-to-agar sampler. Reductions in viable phage concentration ≥3.0 log_10_ (99.9%) were observed after a mean exposure of 3.6 min for Phi6, suggesting efficacy against enveloped viruses (e.g., SARS-CoV-2, influenza, and RSV), and ~10.6 min for MS2, suggesting virucidal efficacy for non-enveloped viruses (e.g., noroviruses and rhinoviruses). This targeted air sanitization approach represents an important non-pharmaceutical public health intervention with virucidal efficacy against airborne viral pathogens.

**IMPORTANCE:**

Airborne viruses are implicated in the transmission indoors of respiratory and enteric viral infections. Air sanitizers represent a non-pharmaceutical intervention to mitigate the risk of such viral transmission. We have developed a method that is now an ASTM International standard (ASTM E3273-21) as well as a test protocol approved by the U.S. EPA to evaluate the efficacy of air sanitizing sprays for inactivating airborne MS2 and Phi6 bacteriophage (used as surrogates for non-enveloped and enveloped human pathogenic viruses, respectively). The test phages were individually suspended in a soil load and aerosolized into a room-sized aerobiology chamber maintained at ambient temperature and relative humidity. Reductions in viable phage concentration ≥3.0 log_10_ (99.9%) were observed after a mean exposure of 3.6 min for Phi6, suggesting efficacy against enveloped viruses (e.g., SARS-CoV-2; influenza; RSV), and ~10.6 min for MS2, suggesting virucidal efficacy for non-enveloped viruses (e.g., noroviruses and rhinoviruses). The data suggest the utility of the air sanitizer for mitigating the risk of indoor viral transmission during viral pandemics and outbreaks.

## INTRODUCTION

The recent SARS-CoV-2 pandemic, as well as the post-pandemic rebounds in case rates for seasonal respiratory and enteric virus infections (1), have heightened the infection prevention and control (IPAC) community’s concerns over the risk of infection from airborne viruses. Infections caused by respiratory viruses, such as SARS-CoV-2, influenzavirus, and respiratory syncytial virus (RSV), as well as enteric viruses, such as noroviruses, are believed to be transmitted from person-to-person directly through contaminated airborne respiratory droplets or aerosols (2). In addition, these viruses may be transmitted indirectly through environmental surfaces contaminated by gravity-induced deposition of respiratory droplets and the subsequent transfer of virus from the contaminated surfaces via hands to the mucous membranes of susceptible human hosts (2).

Investigators continue to debate the relative importance of these different infection transmission pathways even though it is known that there are numerous interdependencies among these (3–8). For instance, the proximate sources of airborne virus droplets (1–5 µm in size) (3–5) are the respiratory discharges from infected persons resulting from coughing, talking, sneezing, expectorating, and so on (4–6). These droplets of varying sizes may evaporate to contribute to infectious viral aerosols depending upon indoor environmental conditions or may settle under the influence of gravity to contaminate environmental surfaces (3–8). Infectious viral aerosols may also be generated through the respiratory discharges mentioned above, through a variety of human activities (e.g., flushing of toilets), or be re-aerosolized by vacuuming or walking on contaminated flooring or by removing contaminated clothing, contributing to airborne virus loads (3–10). Therefore, the possible contributions of the direct and indirect infection transmission pathways should not be discounted, and reduction in airborne virus concentration (quantity of virus per unit volume of air) should mitigate the risk associated with each of the transmission pathways described above.

Air sanitization is just one of the many non-pharmaceutical interventions (NPIs) that may be used to mitigate the infection risk associated with airborne viruses (1, 5, 8, 9, 11–14). The available technologies, including ultraviolet irradiation (9, 11, 15, 16), HEPA filtration or use of portable air purifying devices (11, 17), spray or volatile sanitizer-mediated decontamination (11, 18–26), or radiated microwaves (27), complement other NPI (such as mask wearing) that might be implemented within closed and crowded indoor spaces. Air-sanitization technologies may operate periodically, providing temporary reduction in airborne pathogen load, or may operate continuously, theoretically maintaining airborne pathogen loads to a constant limit. Another aspect of air sanitization technologies is the compatibility of the air sanitization process with human occupancy of the indoor space being sanitized. An optimal air sanitization technology would be compatible with occupancy by humans and would be able to be applied continuously.

The U.S. Environmental Protection Agency (U.S. EPA) released in 2012 guidelines (28) establishing requirements for the assessment of air-sanitization technologies and the generation of in-air microbicidal efficacy data required for a technology’s premarket registration. We have followed these guidelines in evaluating the efficacy of an air sanitizer for inactivating aerosolized *Pseudomonas* phage Phi6 and coliphage MS2 (used as surrogates for human pathogenic viruses). Phi6 is an 80–100 nm enveloped double-stranded RNA phage from the family *Cystoviridae*, which has been used as a surrogate for enveloped viruses such as SARS-CoV-2 in inactivation studies (11, 29–31). MS2 is a ~27 nm non-enveloped single-stranded RNA phage of the *Leviviridae* family which has been used as a surrogate for airborne pathogenic viruses (11, 23, 31–33), and represents a greater in-air virucidal challenge for a candidate air sanitizer than, for instance, the enveloped viruses which cause most of the human respiratory diseases (i.e., orthomyxoviruses such as influenza virus, paramyxoviruses such as respiratory syncytial virus, and coronaviruses such as SARS-CoV-2) (34, 35). Certain non-enveloped viruses cause respiratory illness. These include adenoviruses and rhinoviruses (1). In addition, noroviruses, although enteric viruses, may become airborne, and may be discharged by infected persons in their vomitus or feces. During toilet flushing, the contaminating viruses may become aerosolized, and if the resulting airborne virus is inhaled, the virus may be translocated/swallowed to the target mucous membranes of the gastrointestinal tract to initiate infection (36–38).

In this study, phages Phi6 and MS2 were aerosolized using a six-jet Collison nebulizer in an enclosed room-sized (25 m^3^) aerobiology chamber maintained at 22 ± 2°C and 50 ± 10% relative humidity (RH). Three separate tests were conducted on three manufacturer’s lots of a commercially available 13%-dipropylene glycol (DPG)-containing air sanitizer following EPA’s Guidance for Efficacy Testing at the Lower Certified Limit (LCL) (39). One lot was tested with three replicates for Phi6 and two lots were tested with three replicates for MS2. The resulting virucidal efficacy determinations are reported here.

## MATERIALS AND METHODS

### Challenge bacteriophages, soil load, and viable phage detection system

*Pseudomonas* phage Phi6 (HER 102) was sourced from L’Institut Felix d’Herelle, Université Laval, Quebec, Canada. Coliphage MS2 (ATCC 15597-B1) was sourced from the ATCC through Cedarlane Labs, the Canadian agents for the ATCC. Soil load, which has also been referred to as organic load, is a mixture of organic and inorganic substances intended to mimic human pathophysiological enteric or respiratory body fluids such as mucus, saliva, or fecal matter (40). The phages were suspended in a six-jet Collison nebulizer in phosphate-buffered saline (PBS; MilliporeSigma, USA, pH 7.2 ± 0.2) containing the soil load (40) (0.75 mL of 50 mg/mL bovine serum albumin [BioShop Canada, Canada] stock solution in PBS, 1.05 mL of a 50 mg/mL yeast extract [Fisher Scientific, Canada] solution, 3.0 mL of a 40 mg/mL bovine submaxillary mucin solution [MP Biomedicals, USA], and 10 µL of Antifoam A [MilliporeSigma, USA] to prevent foaming) and 50 µL of MS2 or Phi6 stock in Luria-Bertani (LB) broth (Fisher Scientific, Canada) diluted in PBS (final volume 15 mL) such that the final phage concentrations suspended in the nebulizing vessel would be 4.2–5.0 log_10_ plaque-forming units (PFU)/m^3^ of the aerobiology chamber air.

The bacterial host for Phi6 was *Pseudomonas syringae* subspecies *syringae* (ATCC 19310), while that for MS2 was *Escherichia coli* strain C-3000 (ATCC 15597). Air samples from the chamber were collected directly onto 150 mm diameter Petri plates with separate lawns of the host bacteria. After incubation of the plates, plaques were enumerated to determine the concentrations of the phages in the stocks or residual infectious phages in samples of air collected from the aerobiology chamber, as reported previously (41, 42).

### Testing of the air sanitizer

The air sanitizer tested was Lysol Air Sanitizer (Reckitt, USA), a commercially available ready-to-use indoor air sanitizing spray in pressurized cans and containing 13% DPG (tested at the LCL). At least three containers from each of three separate batches (denoted as 1, 2, and 3) were provided to the testing facility (CREM Co Labs, Canada). At the testing facility, the air sanitizer containers were stored under ambient conditions until used in the aerobiology experiments. A glovebox on one side of the aerobiology chamber allowed access to the inside of the chamber without breaching the containment barrier and enabled operators to release the air sanitizer into the chamber. The outlet of the air sanitizer container was pointed by the operator toward the chamber’s ceiling and spraying was allowed to continue for 30 s while moving the container in a sweeping motion. The air sanitizer container was weighed immediately before and after each release to determine the amount in grams of test agent discharged into the chamber.

### Aerobiology chamber, nebulizer, and sampling device

The aerobiology chamber used in this study has been described previously (41–43). It is a room-sized (25 m^3^ [900 ft^3^] total volume) chamber housed in a clean room with negative pressure with respect to the adjacent areas in a biosafety level 2+ (BSL-2+) containment facility. The chamber was built as specified in the U.S. EPA Guideline (28), which requires a total chamber volume of at least 800 ft^3^ (23 m^3^). Mathematical modeling of the chamber was conducted (41–43) to demonstrate its suitability for use in the experiments described here which were performed according to Good Laboratory Practices (44). Computational fluid dynamics (CFD) was used to optimize different chamber elements such as the speed, angle, and the location of the air circulation fan to have a uniform distribution of the pathogen as well as the spray inside the chamber. It was also established that with the optimized design the air sampled from the center of the chamber represented well the whole chamber. The results from mathematical modeling were also confirmed with actual experiments (41, 43). Furthermore, CFD data were verified by aerobiological experiments carried out for the development of the test protocol. Briefly, the phages under evaluation were suspended in a nebulization medium containing the soil load and aerosolized into the aerobiology chamber using a six-jet Collison nebulizer (CH Technologies, Westwood, NJ, USA) connected to a compressed air cylinder and aerosolization was carried out at a pressure of 1.8 kg/cm^2^. The aerosol size distribution produced by the Collison nebulizer has been reported previously (45). The aerosol was subjected to continuous mixing using a muffin fan (Cooltron AC Fan, Model FA8038B11-7-51, 80 × 80 × 38 mm^3^, 115 VAC, 50/60 Hz, 11/9 W, 26/31 cubic feet per minute [CFM]), and air samples (taken for establishing phage persistence post-aerosolization into the chamber and phage concentration post-air sanitizer use) were collected at pre-determined time periods using a programmable Air Trace slit-to-agar (STA) air sampler (Particle Measuring Systems, USA) (41–43). The air samples were collected from the middle of the aerobiology chamber at a rate of 28.3 L (1 ft^3^) per min and were deposited directly onto *E. coli-* or *P. syringae-*containing lawns in 150 mm diameter LBM (comprised of LB broth + 0.07% lecithin + 0.5% Polysorbate 80) agar plates (41–43). Polysorbate 80 and lecithin were added to the LB broth to neutralize the activity of the active ingredient (DPG) in the air sanitizer.

Between experiments, the aerobiology chamber was flushed with clean air to purge it of any residual aerosolized phage or test air sanitizer. During each experiment, the air temperature (°C) and RH inside the chamber were measured and recorded digitally using a data logger (RTR-500 Datalogger, T&D, Japan).

### Experimental design

The overall study design consisted of three phases: (i) determination of the airborne stability of infectious aerosolized phage in the aerobiology chamber air in the absence of the air sanitizer; (ii) determination of the effectiveness of the neutralizers used to quench the phage inactivation by the air sanitizer; and (iii) examination of the time kinetics of inactivation of viable aerosolized phages in the presence of the air sanitizer and the soil load.

### Evaluation of the airborne stability of the phages in the absence of the air sanitizer

Assessment of phage stability in the aerobiology chamber air following release by the Collison nebulizer was performed before and after each virucidal efficacy testing experiment. The test phages were aerosolized into the aerobiology chamber over a 10-min period. After a 5-min stabilization period to allow the uniform distribution of the phage throughout the chamber’s air, a 2-min baseline air sample was taken to confirm that the protocol requirement for 4.2–5.0 log_10_ PFU/m^3^ of chamber air had been met. Thereafter, 2-min samples of chamber’s air were collected at intervals. The agar plates were incubated at 36 ± 1°C for 18 ± 2 h and then scored for PFU.

### Evaluation of neutralizer effectiveness

The methods used to assess the ability of the LBM agar plates used for air sampling to quench the phage-inactivating activity of the test sanitizer have been described in Supplemental Materials.

### Assessment of test air sanitizer’s virucidal efficacy against aerosolized phage

The virucidal efficacy testing for the batches of air sanitizer was performed against the aerosolized phages, with three independent runs being conducted for each batch. Each run consisted of the following: the test phage was introduced into the aerobiology chamber via the nebulizer over a 10-min period in the presence of a soil load. After a 5-min stabilization period to allow uniform distribution of the phage throughout the chamber air, a 2-min baseline air sample was taken to confirm that the protocol requirement for 4.2–5.0 log_10_ PFU/m^3^ of chamber air had been met. The test air sanitizer was then introduced by spraying directly from the pressurized air sanitizer container into the chamber (through a glove box as explained previously) over a 30-s period. Air samples from the aerobiology chamber were collected at various time points following the spraying of the air sanitizer. The inactivation of airborne phage by the test air sanitizer was effectively quenched at time of sampling onto the LBM agar plates. Air sample plates were incubated at 36 ± 1°C for 18 ± 2 h and then scored for PFU.

## RESULTS

### Stability of viable phage Phi6 aerosolized from a suspending medium containing the soil load in the absence of the air sanitizer

**Fig 1:**
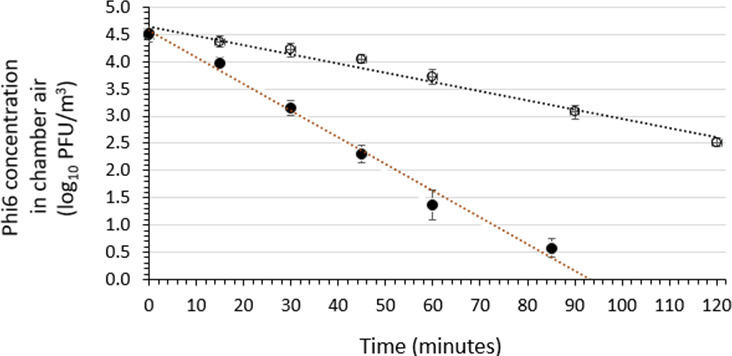
Viability of phage Phi6 aerosolized from suspending medium without and with soil load and in the absence of air sanitizer. Three independent runs were performed for Phi6 in the presence (open symbols) or absence (closed symbols) of soil load. A plot of the average results from the three runs is included, along with the standard deviations. The associated linear regression lines for the average plots are shown in the figure and the equations are *y* = −0.0170 *x* + 4.6504 (*R*² = 0.9727) and *y* = −0.0490 *x* + 4.5665 (*R*² = 0.9887) for Phi6 with soil load and without soil load, respectively.

The stability of viable Phi6 in the aerobiology chamber air over a 120-min period following introduction through the Collison nebulizer in the presence or absence of the soil load and absence of air sanitizer was determined in three independent runs each (Fig. 1). This control testing was performed at 22 ± 2°C and 50 ± 10% RH. The results indicate log-linear (first order) Phi6 viability decay over time. The average decay rates in the presence and absence of the soil load were determined to be 0.0170 log_10_ PFU/m^3^/min and 0.0490 log_10_ PFU/m^3^/min, respectively. The natural decay rate for Phi6 was much higher in the absence than in the presence of the soil load, such that at the end of 120 min, the Phi6 concentration in the presence of soil load was 2.5 log_10_ PFU/m^3^. Viable Phi6 in the absence of the soil load could not be detected in the chamber air after about 90 min.

### Stability of viable phage MS2 aerosolized from a suspending medium containing the soil load in the absence of the air sanitizer

**Fig 2:**
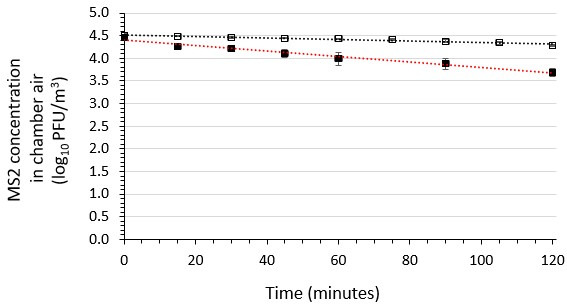
Viability of phage MS2 aerosolized from suspending medium containing the soil load in aerobiology chamber’s air samples in the absence of the air sanitizer. Three independent runs were performed for MS2 in the presence (open symbols) or absence (closed symbols) of a soil load. A plot of the average results from the three runs is shown, along with the standard deviations. The associated linear regression lines for the plots are shown and the equations are *y* = −0.0026 *x* + 4.2025 (*R*² = 0.8964) and *y* = −0.0060 *x* + 4.3952 (*R*² = 0.9721) for MS2 with soil load and without soil load, respectively.

The viability of MS2 in the aerobiology chamber’s air over a 120-min period following introduction through the Collison nebulizer in the presence or absence of the soil load but in the absence of the air sanitizer was determined in three independent runs each (Fig. 2). This control testing was performed at 22 ± 2°C and 50 ± 10% RH. The results indicate a log-linear (first order) decay in aerobiology chamber air over time. The average decay rates in the presence and absence of soil load were determined to be 0.0026 log_10_ PFU/m^3^/min and 0.0060 log_10_ PFU/m^3^/min, respectively. The natural decay rate for MS2 was greater in the absence than in the presence of soil load, such that at the end of 120 min, the MS2 concentration in the presence of soil load was 0.5 log_10_ higher than in the absence of soil load.

### Evaluation of neutralization of the phage-inactivating activity in LBM agar

The results of the evaluation of the ability of the LBM agar plates used for aerobiology chamber air sampling to quench the phage-inactivating activity of the test air sanitizer have been described in Supplemental Materials. The evaluation was performed separately for batches 1 and 2 of test air sanitizer.

### Assessment of the test air sanitizer’s efficacy for inactivating aerosolized phage Phi6

**Fig 3:**
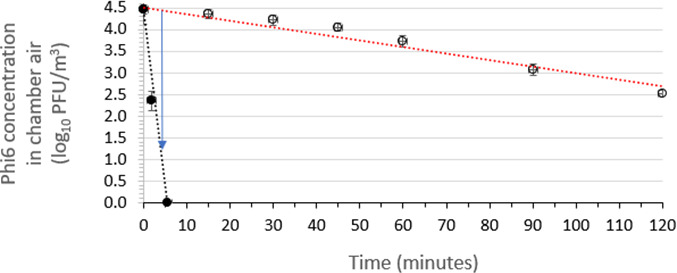
Time kinetics for virucidal efficacy of air sanitizer batch 1 for inactivating aerosolized phage Phi6 suspended in medium containing soil load in a room-sized aerobiology chamber. The red dotted line displays the linear regression line fit to the Phi6 concentrations in the absence of air sanitizer (open symbols), while the black dotted line displays the linear regression line fit to the Phi6 concentrations in the presence of air sanitizer (closed symbols). The blue arrow indicates the time required to achieve 3 log_10_ reduction of Phi6 concentration in chamber air. All data points indicate mean ± standard deviation (*n* = 3 independent runs).

The time kinetics of reduction of viable aerosolized Phi6 in the aerobiology chamber’s air over a 120-min period following a 30-s spraying of the test air sanitizer were determined in three independent runs for a single air sanitizer batch. This efficacy testing was performed at 22 ± 2°C and 50 ± 10% RH in the presence of soil load. The results for air sanitizer batch 1 are displayed in Fig. 3. The plot of inactivation versus time post-spraying for the air sanitizer batch is linear. The linear regression line fits for the average efficacy points and the average control points are displayed in Fig. 3. The average efficacy curve and average control curve were fitted with a linear regression line and the time required for achieving a 3-log_10_ reduction in viable Phi6 concentration was calculated by subtracting the average efficacy curve from the average control curve. A 3.0 log_10_ reduction in viable Phi6 concentration was achieved within 3.6 min of air sanitizer release.

### Assessment of the air sanitizer’s efficacy for inactivating aerosolized non-enveloped coliphage MS2

**Fig 4:**
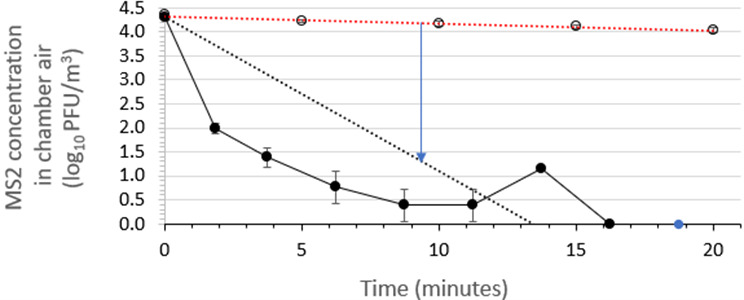
Time kinetics for virucidal efficacy of air sanitizer batch 2 for reducing the viability of aerosolized phage MS2 suspended in medium containing soil load in a room-sized aerobiology chamber. The red dotted line displays the linear regression line fit to the MS2 concentrations in the absence of air sanitizer (open symbols), while the black dotted line displays the linear regression line fit to the MS2 concentrations in the presence of air sanitizer (closed symbols). The blue arrow indicates the time required to achieve a 3 log_10_ reduction in MS2 concentration in chamber air. All data points indicate mean ± standard deviation (*n* = 3 independent runs). The spike in the MS2 concentration plot at ~14 min is considered to be an anomaly and cannot be readily explained.

**Fig 5:**
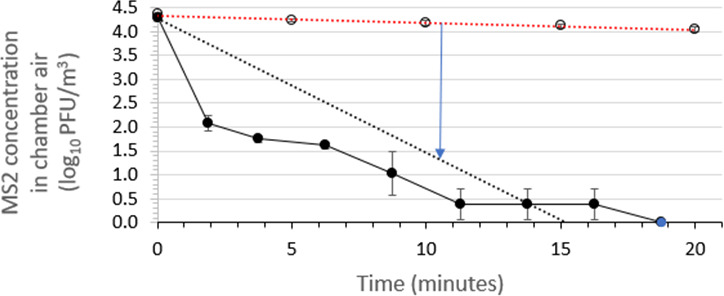
Time kinetics for virucidal efficacy of air sanitizer batch 3 for reducing the viability of aerosolized phage MS2 suspended in soil load in a room-sized aerobiology chamber. The red dotted line displays the linear regression line fit to the MS2 concentrations in the absence of air sanitizer (open symbols), while the black dotted line displays the linear regression line fit to the MS2 concentrations in the presence of air sanitizer (closed symbols). The blue arrow indicates the time required to achieve a 3 log_10_ reduction in MS2 concentration in chamber air. All data points indicate mean ± standard deviation (*n* = 3 independent runs).

The time kinetics of inactivation of airborne viable MS2 in the aerobiology chamber air over a 10-min period following a 30-s spraying of the test air sanitizer were determined in three independent runs per air sanitizer batch. This efficacy testing was performed at 22 ± 2°C and 50 ± 10% RH. The results for air sanitizer batch 2 are displayed in Fig. 4, while the results for batch 3 are shown in Fig. 5. The plots of inactivation versus time post-spraying for the two air sanitizer batches each exhibited an initial steep portion followed by a more linear terminal portion.

The linear regression line fits for the average efficacy points as the average control points are displayed in Fig. 4 and 5. These regression lines were forced through the *y*-axis intercept values informed by the average time_0_ MS2 concentrations determined for the set of experiments performed for each air sanitizer batch (4.28 log_10_ PFU/m^3^ for batch 2 and 4.31 log_10_ PFU/m^3^ for batch 3). The time required for achieving a 3-log_10_ reduction in viable MS2 concentration was calculated by subtracting the average efficacy curve from the average control curve as displayed in Fig. 4 and 5. These average times were found to be 11.3 min for batch 2 and 9.8 min for batch 3. As is evident from Fig. 4 and 5, the approach used for determining time post-spraying required to achieve a 3-log_10_ reduction in airborne viable MS2 concentration within the aerobiology chamber is quite conservative. This provides a safety factor and appears justified, considering the non-linearity of the time post-spraying versus viable MS2 concentration curves.

## DISCUSSION

A methodology for demonstrating the virucidal efficacy of air sanitizers for inactivating airborne pathogens was first incorporated into an aerobiology protocol developed by the authors per U.S. EPA Guidelines (28) published in 2012. Since then, the method has been approved by the U.S. EPA and the Association of Home Appliance Manufacturers (AHAM) (46) and is now the subject of an ASTM International standard (47). Prior to this, a pathway for testing and U.S. EPA registration of an air-sanitizing product intended for room air decontamination did not exist (31). The 2012 U.S. EPA Guidelines (28) address “testing to demonstrate the effectiveness of antimicrobial pesticides bearing label claims for the treatment of air to temporarily reduce the number of airborne bacteria.” The distinction imparted by “temporarily reduce” is important, as it is understood that the typical air-sanitization product will only be effective over a finite period of time following application. In the presence of a pathogen source, such as an infected person (including symptomatic, pre-symptomatic, and asymptomatic), recontamination of the indoor air will be inevitable.

The 2012 U.S. EPA Guidelines (28) do not address the efficacy of air sanitizers for inactivating airborne viruses. Using the principles outlined in the guidelines, the authors developed an aerovirological protocol to determine virus stability in the absence of an air sanitizer and virucidal efficacy post-exposure to air sanitizer under ambient environmental conditions in a room-sized aerobiology chamber for registration of air sanitizer with claims for in-air virucidal and bactericidal activity (42). In the present work, we have used bacteriophage Phi6 as a surrogate for enveloped respiratory viruses, such as the coronavirus SARS-CoV-2, the orthomyxovirus influenza virus, and the pneumovirus RSV), and MS2 as a surrogate for non-enveloped airborne respiratory and enteric viruses of human concern (such as rhinoviruses, noroviruses, and adenoviruses). The use of phages as surrogates for human pathogenic viruses in aerovirological investigations has been reported previously (11, 23, 29–33).

Bacteriophage MS2 (required by the EPA-approved protocol), is a small (~27 nm), non-enveloped phage that represents a worst-case challenge for most formulated microbicidal actives and many physical inactivation approaches. According to the principle of the hierarchy of pathogens to microbicidal actives (34, 35), the lack of a lipid envelope is expected to pose a greater challenge to the inactivating effects of several classes of microbicides. One might argue, therefore, that challenging an air sanitizer intended for use against enveloped viruses, such as the coronaviruses including SARS-CoV-2, with a non-enveloped virus such as MS2 is overly stringent (Fig. 3 vs. Fig. 4 and 5). Nevertheless, the results from the present study suggest that the air sanitizer evaluated will be effective against a broad variety of respiratory viruses, both enveloped (e.g., influenza viruses, RSV, and coronaviruses including SARS-CoV-2) and non-enveloped (e.g., adenoviruses and noroviruses).

The experimental design followed in this study incorporated a room-sized aerobiology chamber, but also included certain aspects rendering the airborne viral inactivation challenge even greater (42). For instance, the inclusion of the soil load (40) in the inactivation matrix represents an additional level of stringency to an air sanitizer targeting airborne viruses. The soil load provides not only a field-relevant organic/inorganic matrix, but also an additional challenge for the test air sanitizer to overcome during virucidal efficacy testing of the targeted aerosolized viruses. Another factor that increases the stringency of the present aerobiology study is the use of a programmable STA air sampler (26). The STA type of air sampler has been found to be more effective than liquid impingers used in aerovirological investigation (23, 48). This type of air sampler enables aerosolized virus to be captured directly in real time on semi-solid agar containing a lawn of host bacterial cells, and following the subsequent incubation period, to be enumerated as lytic plaques produced by residual viable phage (42).

The stability (retention of viability/infectivity) of aerosolized Phi6 and MS2 was established in the absence of exposure to the air-sanitizing agent in the presence or absence of the soil load. As mentioned, the tripartite soil load consists of tryptone or yeast, bovine serum albumin, and bovine mucin (40) and is intended to mimic the pathophysiological matrices with which viruses are typically associated when released into the environment. Our data demonstrate that the soil load stabilizes the infectivity of aerosolized Phi6 (the enveloped phage) (infectivity decay rate in the absence of the soil load = 0.0490 log_10_ PFU/m^3^/min; decay rate in the presence of soil load = 0.0170 log_10_ PFU/m^3^/min [Fig. 1]). The stabilizing effect of the soil load on aerosolized MS2 (the non-enveloped phage) was less striking, but still evident (infectivity decay rate in absence of the soil load = 0.0060 log_10_ PFU/m^3^/min; decay rate in presence of soil load = 0.0026 log_10_ PFU/m^3^/min [Fig. 2]). This suggests that there are phage viability-stabilizing components in the soil load. This finding is consistent with the protection of aerosolized influenza A virus in the presence of a protein load (human bronchial epithelial extracellular matrix [HBE ECM]) reported previously by Kormuth et al. (49). In their study, the protection was observed in all RH levels evaluated and was HBE ECM concentration dependent.

The protection of aerosolized viruses may depend on the nature of the soil load, specifically the presence of protein and carbohydrate components. Proteins and mucin glycoproteins in virus-suspending medium have been proposed to form a protective layer on aerosolized viruses at different temperatures and RH (50–52). It previously has been reported (53) that when human coronavirus 229E (HCoV 229E) and poliovirus type 1 (Sabin) are co-aerosolized as mixtures suspended in medium containing Tryptose phosphate broth (which contains Tryptose, dextrose, sodium chloride, and disodium phosphate at pH 7.3), HCoV 229E infectivity persists longest at 20°C and 50% and 30% RH. Furthermore, at 20°C and 50% RH, 20% of the residual viable HCoV 229E may still be recovered at 6 days of viable aerosol age (53). However, when infectious HCoV 229E is aerosolized at a lower temperature (6°C), the reverse has been observed (that is, the virus survived equally well at 80% RH, in addition to 30% RH and 50% RH) (53). That viable aerosolized HCoV 229E survives equally well from low to high RH at low temperature suggests that the viscoelasticity provided by the protein and carbohydrate matrix potentially vitrifies or crystalizes the external viral proteins, thereby stabilizing the aerosolized viable virus and providing protection of the structural proteins (host cell receptor-binding proteins) required for virus-host interactions leading to initiation of infection of a susceptible person’s mucus membrane. When poliovirus was evaluated in the same set of experimental conditions (53) (20°C and 30%, 50%, and 80% RH) and co-aerosolized with HCoV 229E, the poliovirus survived only at the high (80%) RH. This represented a striking difference between the enveloped HCoV 229E and the non-enveloped poliovirus and confirmed the stability of aerosolized HCoV 229E (enveloped virus) and poliovirus (non-enveloped virus) when investigated under the identified set of environmental conditions (aerosolized as a mixture of the two viruses) and reported previously (53, 54). This finding has also been reported recently for the betacoronavirus, mouse hepatitis virus (MHV) (52), particularly when the aerosolized MHV was investigated at low RH. Thus, the stability of viruses in aerosols appears to be more complex than previously thought, depending upon the interplay of temperature, RH, and the nature of the virus-suspending medium (including the organic matrix used for virus aerosolization), as observed in the present investigation (Fig. 1).

Review of the literature reveals that several investigations of the virucidal efficacy of physical and microbicidal approaches for inactivating aerosolized viruses have been conducted (8, 9, 11, 13–27, 31, 33, 55). The advantages and disadvantages of the various physical approaches are considered out of scope for the present article. Candidate microbicidal agents have included ozone (31), hydrogen peroxide (18, 19, 26), chlorine dioxide (55), and glycols, including triethylene glycol (20–23), propylene glycol (24, 25), and DPG (26). The evaluation of the glycols for this application stems in part from their relatively low toxicity to humans (56–58). Desai et al. (23) investigated the efficacy of a commercially available formulation of triethylene glycol (TEG) for inactivating MS2 bacteriophage in 15.3 and 21.6 m^3^ aerobiology chambers run at 23–26°C and 30–40% RH. Samples of air from the chamber were collected using either Biosamplers or AGI-30 samplers. The authors (23) reported that 3-log_10_ reductions in MS2 titer, accounting for “natural settling [no mixing of the chamber air was reported] and die-off” of the MS2 were achieved within 15 min of application of 0.287 mg/m^3^ TEG. Gomez et al. (26) evaluated the efficacy of a commercially available formulated DPG-containing spray product for inactivating MHV, a surrogate for SARS-CoV-2 (enveloped, family *Coronaviridae*). The studies involved a 9-m^3^ aerobiology chamber at 22°C and 60% RH and air mixing. Sampling was via time-resolved condensation growth tube capture sampler. The time required for <20 ppm DPG/ethanol to achieve a 2-log_10_ reduction in MHV titer was reported as 33 ± 15 min (26). Methodological differences between the Desai et al. (23) and Gomez et al. (26) studies and the current study therefore included: use of different aerobiology chamber volumes, testing of enveloped versus non-enveloped viruses, implementation of air mixing, presence or absence of soil load simulating a patient’s pathophysiological secretions, air sampler type, and time points sampled. These factors have now been dictated in the aerobiology protocol followed in the present study. This protocol has now been incorporated into an ASTM International Standard (47), enabling standardization of methods for future studies of this kind.

The virucidal activity of the DPG-containing air sanitizer evaluated in this study may result from impacts on the receptor-binding domain of the viral envelope or capsid and alterations in virus-host interactions, hindering virus attachment to the host cell membrane and, subsequently, fusion and penetration by enveloped viruses and non-enveloped viruses (59–62). The exact mechanism of virucidal activity of DPG against aerosolized enveloped and non-enveloped viruses requires further investigation.

To our knowledge, the leading air-fresheners (fragrances used to mask unpleasant odors) commercially available in the United States have not been assessed for their bactericidal and virucidal activities in indoor air spaces using the standardized protocol (28, 47) used for the aerovirological investigations reported here. This reflects, in part, the previous lack of a specific aerobiology protocol, as well as lack of capability of testing facilities for conducting field-relevant aerobiology studies in indoor air spaces (especially, room-sized aerobiology chambers). The results of the current study enabled the U.S. EPA to provide the first registration for an air sanitizer (58). This registration was awarded not only on the basis of the virucidal efficacy data discussed in this paper, but also on data on bactericidal efficacy (63), suppression of odors, and recognized safety for human health and the environment in both household and commercial settings (58). Air-sanitizing products are not intended to replace the Centers for Disease Control and Prevention, state, and local public health guidelines around NPIs, such as mask wearing, social distancing, surface hygiene, hand sanitization, and so on (58). Rather, a holistic interventional approach, focusing on both contaminated surfaces and air, for mitigating risk of indoor virus transmission is advocated by the authors (8).

### Conclusions

Respiratory viruses and certain enteric viruses can be transmitted via indoor air, either directly (in aerosols or droplets) or indirectly through contamination of environmental surfaces and subsequent infection of susceptible humans via re-aerosolization from contaminated surfaces or by contacting contaminated surfaces with hands (8). Sanitization of indoor air would, therefore, appear to represent an important NPI for mitigating infection risk from airborne viruses. This intervention may be particularly useful during pandemics (such as the COVID-19/SARS-CoV-2 pandemic) and possible future pandemics [e.g., involving Nipah virus (64)] or seasonal virus outbreaks, and in high-risk indoor situations including overcrowding or the presence of pre-symptomatic, symptomatic, or asymptomatic virus-infected patients. Like most air sanitization approaches, indoor spaces should not be occupied during the application of air sanitizers, and reapplication will be needed periodically as the indoor air becomes reinfected by a virus-shedding person. Proper formulation of an air sanitizer containing DPG has been shown to lead to an EPA-registered air sanitizer which causes rapid (10.6 min) and effective (≥3 log_10_) reduction in the concentration of viable airborne non-enveloped phage MS2 (a surrogate for non-enveloped viruses). An even shorter time (3.6 min) was required to inactivate 3 log_10_ of the enveloped phage Phi6. By inference, the delivery system incorporating the formulated DPG active ingredient evaluated in this study should be effective against any enveloped viruses (most respiratory viruses are enveloped, including coronaviruses such as SARS-CoV-2, influenza, and respiratory syncytial virus) or non-enveloped viruses (including adenoviruses and noroviruses). Additional work may be performed to extend this finding to enveloped vertebrate viruses of human health concern (influenza virus, paramyxoviruses, coronaviruses including SARS-CoV-2). This may allow the contact time required to be reduced in the case of air sanitizers targeting specific enveloped viruses (i.e., less contact time may be required for inactivating enveloped respiratory viruses than for the MS2 phage evaluated in this study as required by the U.S. EPA to substantiate the current virucidal efficacy claims).

## Data Availability

Data are provided within the article and supplemental material.

## SUPPLEMENTAL MATERIAL

Supplemental material aem.01426-24-s0001.pdf Methods and results for neutralizing ability.

ASM does not own the copyrights to Supplemental Material that may be linked to, or accessed through, an article. The authors have granted ASM a non-exclusive, world-wide license to publish the Supplemental Material files. Please contact the corresponding author directly for reuse.

## References

[1] Ijaz MK, Nims RW, Rubino JR, McKinney J, Gerba CP. 2022. Lessons learned from the SARS-CoV-2 pandemic: preparing for the next outbreak of respiratory and enteric viral infections. Appl Microbiol Open Access 8:229. doi:10.35284/2471-9315.22.8.229

[2] Yezli S, Otter JA. 2011. Minimum infective dose of the major human respiratory and enteric viruses transmitted through food and the environment. Food Environ Virol 3:1–30. doi:10.1007/s12560-011-9056-7 35255645 PMC7090536

[3] Kutter JS, Spronken MI, Fraaij PL, Fouchier RA, Herfst S. 2018. Transmission routes of respiratory viruses among humans. Curr Opin Virol 28:142–151. doi:10.1016/j.coviro.2018.01.001 29452994 PMC7102683

[4] Zhang XS, Duchaine C. 2020. SARS-CoV-2 and health care worker protection in low-risk settings: a review of modes of transmission and a novel airborne model involving inhalable particles. Clin Microbiol Rev 34:e00184-20. doi:10.1128/CMR.00184-20 33115724 PMC7605309

[5] Morawska L, Johnson GR, Ristovski ZD, Hargreaves M, Mengersen K, Corbett S, Chao CYH, Li Y, Katoshevski D. 2009. Size distribution and sites of origin of droplets expelled from the human respiratory tract during expiratory activities. J Aerosol Sci 40:256–269. doi:10.1016/j.jaerosci.2008.11.002 PMC7126899 32287373

[6] Ram K, Thakur RC, Singh DK, Kawamura K, Shimouchi A, Sekine Y, Nishimura H, Singh SK, Pavuluri CM, Singh RS, Tripathi SN. 2021. Why airborne transmission hasn’t been conclusive in case of COVID-19? An atmospheric science perspective. Sci Total Environ 773:145525. doi:10.1016/j.scitotenv.2021.145525 33940729 PMC7984961

[7] Leung NHL. 2021. Transmissibility and transmission of respiratory viruses. Nat Rev Microbiol 19:528–545. doi:10.1038/s41579-021-00535-6 33753932 PMC7982882

[8] Ijaz MK, Sattar SA, Nims RW, Boone SA, McKinney J, Gerba CP. 2023. Environmental dissemination of respiratory viruses: dynamic interdependencies of respiratory droplets, aerosols, aerial particulates, environmental surfaces, and contribution of viral re-aerosolization. PeerJ 11:e16420. doi:10.7717/peerj.16420 38025703 PMC10680453

[9] Ijaz MK, Zargar B, Wright KE, Rubino JR, Sattar SA. 2016. Generic aspects of the airborne spread of human pathogens indoors and emerging air decontamination technologies. Am J Infect Control 44:S109–S120. doi:10.1016/j.ajic.2016.06.008 27590695 PMC7115269

[10] Samet JM, Prather K, Benjamin G, Lakdawala S, Lowe J-M, Reingold A, Volckens J, Marr LC. 2021. Airborne transmission of severe acute respiratory syndrome coronavirus 2 (SARS-CoV-2): what do we know. Clin Infect Dis 73:1924–1926. doi:10.1093/cid/ciab039 33458756 PMC7929061

[11] Duchaine C. 2016. Assessing microbial decontamination of indoor air with particular focus on human pathogenic viruses. Am J Infect Control 44:S121–S126. doi:10.1016/j.ajic.2016.06.009 27590696 PMC7115274

[12] Wei J, Li Y. 2016. Airborne spread of infectious agents in the indoor environment. Am J Infect Control 44:S102–S108. doi:10.1016/j.ajic.2016.06.003 27590694 PMC7115322

[13] Allen RW, Barn P. 2020. Individual- and household-level interventions to reduce air pollution exposures and health risks: a review of recent literature. Curr Environ Health Rep 7:424–440. doi:10.1007/s40572-020-00296-z 33241434 PMC7749091

[14] Song L, Zhou J, Wang C, Meng G, Li Y, Jarin M, Wu Z, Xie X. 2022. Airborne pathogenic microorganisms and air cleaning technology development: a review. J Hazard Mater 424:127429. doi:10.1016/j.jhazmat.2021.127429 34688006

[15] Memarzadeh F. 2021. A review of recent evidence for utilizing ultraviolet irradiation technology to disinfect both indoor air and surfaces. Appl Biosaf 26:52–56. doi:10.1089/apb.20.0056 36033964 PMC8869636

[16] Walker CM, Ko G. 2007. Effect of ultraviolet germicidal irradiation on viral aerosols. Environ Sci Technol 41:5460–5465. doi:10.1021/es070056u 17822117

[17] Rodríguez M, Palop ML, Seseña S, Rodríguez A. 2021. Are the portable air cleaners (PAC) really effective to terminate airborne SARS-CoV-2? Sci Total Environ 785:147300. doi:10.1016/j.scitotenv.2021.147300 33940414 PMC8081570

[18] Hislop M, Grinstead F, Henneman JR. 2022. Hybrid hydrogen peroxide for viral disinfection. In Nims RW, Ijaz MK (ed), Disinfection of Viruses. Intech Open, London, UK.

[19] Lee C, Henneman JR. 2022. Dry hydrogen peroxide for viral inactivation. In Nims RW, Ijaz MK (ed), Disinfection of viruses. Intech Open, London, UK.

[20] Sahyun M. 1948. Air disinfection by triethylene glycol vapor. Stanford Med Bull 6:457–468. 18121733

[21] Krugman S, Ward R. 1951. Air sterilization in an infants’ ward; effect of triethylene glycol vapor and dust-suppressive measures on respiratory cross infection rate. J Am Med Assoc 145:775–780. doi:10.1001/jama.1951.02920290001001 14803254

[22] Goldman E, Choueiri TK, Mainelis G, Ramachandran G, Schaffner DW. 2022. Triethylene glycol can be predeployed as a safe virus-killing indoor air treatment. J Infect Dis 226:2040–2041. doi:10.1093/infdis/jiac39436177834

[23] Desai G, Ramachandran G, Goldman E, Esposito W, Galione A, Lal A, Choueiri TK, Fay A, Jordan W, Schaffner DW, Caravanos J, Grignard E, Mainelis G. 2023. Efficacy of Grignard Pure to inactivate airborne phage MS2, a common SARS-CoV-2 surrogate. Environ Sci Technol 57:4231–4240. doi:10.1021/acs.est.2c08632 36853925 PMC10001433

[24] Styles CT, Zhou J, Flight KE, Brown JC, Vanden Oever M, Peacock TP, Wang Z, Millns R, O’Neill JS, Barclay WS, Tregoning JS, Edgar RS. 2023. Propylene glycol inactivates respiratory viruses and prevents airborne transmission. bioRxiv. doi:10.1101/2023.02.13.528349 PMC10701621 37970627

[25] Hirama Y, Onishi S, Shibata R, Ishida H, Mori T, Ota N. 2023. Antiviral effect of propylene glycol against envelope viruses in spray and volatilized forms. Viruses 15:1421. doi:10.3390/v15071421 37515109 PMC10385749

[26] Gomez O, McCabe KM, Biesiada E, Volbers B, Kraus E, Nieto-Caballero M, Hernandez M. 2022. Airborne murine coronavirus response to low levels of hypochlorous acid, hydrogen peroxide and glycol vapors. Aerosol Sci Technol 56:1047–1057. doi:10.1080/02786826.2022.2120794

[27] Manna A, De Forni D, Bartocci M, Pasculli N, Poddesu B, Lista F, De Santis R, Amatore D, Grilli G, Molinari F, Sangiovanni Vincentelli A, Lori F. 2023. SARS-CoV-2 inactivation in aerosol by means of radiated microwaves. Viruses 15:1443. doi:10.3390/v15071443 37515131 PMC10386662

[28] U.S. Environmental Protection Agency. 2012. OCSPP 810. 2500-Air Sanitizers_2012-03-12 [EPA 730-C-11-003]. Available from: https://www.regulations.gov/document/EPA-HQ-OPPT-2009-0150-0025

[29] Fedorenko A, Grinberg M, Orevi T, Kashtan N. 2020. Survival of the enveloped bacteriophage Phi6 (a surrogate for SARS-CoV-2) in evaporated saliva microdroplets deposited on glass surfaces. Sci Rep 10:22419. doi:10.1038/s41598-020-79625-z 33376251 PMC7772334

[30] Lin K, Schulte CR, Marr LC. 2020. Survival of MS2 and Φ6 viruses in droplets as a function of relative humidity, pH, and salt, protein, and surfactant concentrations. PLoS ONE 15:e0243505. doi:10.1371/journal.pone.0243505 33290421 PMC7723248

[31] Dubuis M-E, Dumont-Leblond N, Laliberté C, Veillette M, Turgeon N, Jean J, Duchaine C. 2020. Ozone efficacy for the control of airborne viruses: bacteriophage and norovirus models. PLoS ONE 15:e0231164. doi:10.1371/journal.pone.0231164 32275685 PMC7147755

[32] Turgeon N, Toulouse M-J, Martel B, Moineau S, Duchaine C. 2014. Comparison of five bacteriophages as models for viral aerosol studies. Appl Environ Microbiol 80:4242–4250. doi:10.1128/AEM.00767-14 24795379 PMC4068686

[33] Chen L, Lee W-J, Ma Y, Jang SS, Fong K, Wang S. 2022. The efficacy of different sanitizers against MS2 bacteriophage introduced onto plastic or stainless steel surfaces. Curr Res Food Sci 5:175–181. doi:10.1016/j.crfs.2022.01.004 35072105 PMC8761864

[34] Sattar SA. 2007. Hierarchy of susceptibility of viruses to environmental surface disinfectants: a predictor of activity against new and emerging viral pathogens. J AOAC Int 90:1655–1658. doi:10.1093/jaoac/90.6.1655 18193744

[35] Ijaz MK, Sattar SA, Rubino JR, Nims RW, Gerba CP. 2020. Combating SARS-CoV-2: leveraging microbicidal experiences with other emerging/re-emerging viruses. PeerJ 8:e9914. doi:10.7717/peerj.9914 33194365 PMC7485481

[36] Alsved M, Fraenkel C-J, Bohgard M, Widell A, Söderlund-Strand A, Lanbeck P, Holmdahl T, Isaxon C, Gudmundsson A, Medstrand P, Böttiger B, Löndahl J. 2020. Sources of airborne norovirus in hospital outbreaks. Clin Infect Dis 70:2023–2028. doi:10.1093/cid/ciz584 31257413 PMC7201413

[37] Xiao S, Tang JW, Li Y. 2017. Airborne or fomite transmission for norovirus? A case study revisited. Int J Environ Res 14:1571. doi:10.3390/ijerph14121571 PMC5750989 29240665

[38] Boles C, Brown G, Nonnenmann M. 2021. Determination of murine norovirus aerosol concentration during toilet flushing. Sci Rep 11:23558. doi:10.1038/s41598-021-02938-0 34876637 PMC8651634

[39] U.S. Environmental Protection Agency. 2023. Guidance for efficacy testing at the lower certified limits. Available from: https://www.epa.gov/pesticide-registration/guidance-efficacy-testing-lower-certified-limits

[40] Springthorpe VS, Sattar SA. 2007. Application of a quantitative carrier test to evaluate microbicides against mycobacteria. J AOAC Int 90:817–824. doi:10.1093/jaoac/90.3.817 17580635

[41] Zargar B, Kashkooli FM, Soltani M, Wright KE, Ijaz MK, Sattar SA. 2016. Mathematical modeling and simulation of bacterial distribution in an aerobiology chamber using computational fluid dynamics. Am J Infect Control 44:S127–37. doi:10.1016/j.ajic.2016.06.005 27590697

[42] Zargar B, Sattar SA, Kibbee R, Rubino J, Ijaz MK. 2022. Direct and quantitative capture of viable bacteriophages from experimentally contaminated indoor air: a model for the study of airborne vertebrate viruses including SARS-CoV-2. J Appl Microbiol 132:1489–1495. doi:10.1111/jam.15262 34411388 PMC8447128

[43] Kashkooli FM, Soltani M, Zargar B, Ijaz MK, Taatizadeh E, Sattar SA. 2020. Analysis of an indoor air decontamination device inside an aerobiology chamber: a numerical-experimental study. Air Qual Atmos Health 13:281–288. doi:10.1007/s11869-019-00782-w

[44] U.S. Food and Drug Administration. Title 21 code of federal regulations part 58. Good laboratory practice for nonclinical laboratory studies. Available from: https://www.accessdata.fda.gov/scripts/cdrh/cfdocs/cfcfr/CFRSearch.cfm?CFRPart=58

[45] Ijaz MK, Karim YG, Sattar SA, Johnson-Lussenburg CM. 1987. Development of methods to study the survival of airborne viruses. J Virol Methods 18:87–106. doi:10.1016/0166-0934(87)90114-5 2828403 PMC7119592

[46] Association of Home Appliance Manufacturers. 2022. AHAM publishes first standard to measure room air cleaner ability to remove viruses and bacteria from indoor air. Available from: https://www.aham.org/AHAM/News/Latest_News/New_Air_Cleaner_Standard_Measures_Virus_Removal.aspx

[47] ASTM International. 2021. ASTM E3273-21. Standard practice to assess microbial decontamination of indoor air using an aerobiology chamber

[48] Borges JT, Nakada LYK, Maniero MG, Guimarães JR. 2021. SARS-CoV-2: a systematic review of indoor air sampling for virus detection. Environ Sci Pollut Res Int 28:40460–40473. doi:10.1007/s11356-021-13001-w 33630259 PMC7905194

[49] Kormuth KA, Lin K, Prussin AJ 2nd, Vejerano EP, Tiwari AJ, Cox SS, Myerburg MM, Lakdawala SS, Marr LC. 2018. Influenza virus infectivity is retained in aerosols and droplets independent of relative humidity. J Infect Dis 218:739–747. doi:10.1093/infdis/jiy221 29878137 PMC6057527

[50] Chatterjee M, van Putten JPM, Strijbis K. 2020. Defensive properties of mucin glycoproteins during respiratory infections – relevance for SARS-CoV-2. MBio 11:e02374-20. doi:10.1128/mBio.02374-20 33184103 PMC7663010

[51] Chatterjee M, Huang LZX, Mykytyn AZ, Wang C, Lamers MM, Westendorp B, Wubbolts RW, van Putten JPM, Bosch B-J, Haagmans BL, Strijbis K. 2023. Glycosylated extracellular mucin domains protect against SARS-CoV-2 infection at the respiratory surface. PLoS Pathog 19:e1011571. doi:10.1371/journal.ppat.1011571 37561789 PMC10464970

[52] Nieto-Caballero M, Davis RD, Fuques E, Gomez OM, Huynh E, Handorean A, Ushijima S, Tolbert M, Hernandez M. 2023. Carbohydrate vitrification in aerosolized saliva is associated with the humidity-dependent infectious potential of airborne coronavirus. PNAS Nexus 2:pgac301. doi:10.1093/pnasnexus/pgac301 PMC9896139 36743472

[53] Ijaz MK, Brunner AH, Sattar SA, Nair RC, Johnson-Lussenburg CM. 1985. Survival characteristics of airborne human coronavirus 229E. J Gen Virol 66 (Pt 12):2743–2748. doi:10.1099/0022-1317-66-12-2743 2999318

[54] Sattar SA, Ijaz MK. 1987. Spread of viral infections by aerosols. Crit Rev Environ Control 17:89–131. doi:10.1080/10643388709388331

[55] Jones S, Lukasik G, Driver J, Harbison R. 2022. Virucidal efficacy of chlorine dioxide interventions on MS2 phage bioaerosol in a laboratory chamber. Occup Dis Environ Med 10:206–216. doi:10.4236/odem.2022.103016

[56] Fowles JR, Banton MI, Pottenger LH. 2013. A toxicological review of the propylene glycols. Crit Rev Toxicol 43:363–390. doi:10.3109/10408444.2013.792328 23656560

[57] U.S. Environmental Protection Agency. 2006. Reregistration eligibility decision for propylene glycol and dipropylene glycol. [EPA-739-R-06-002\.206]. Available from: https://www3.epa.gov/pesticides/chem_search/reg_actions/reregistration/red

[58] U.S. Environmental Protection Agency. 2023. EPA registers air sanitizer for residential and commercial use against influenza and coronavirus. Available from: https://www.epa.gov/pesticides/epa-registers-air-sanitizer-residential-and-commercial-use-against-influenza-and

[59] Pletan ML, Tsai B. 2022. Non-enveloped virus membrane penetration: new advances leading to new insights. PLoS Pathog 18:e1010948. doi:10.1371/journal.ppat.1010948 36480535 PMC9731489

[60] Li X, Yuan H, Li X, Wang H. 2023. Spike protein mediated membrane fusion during SARS‐CoV‐2 infection. J Med Virol 95. doi:10.1002/jmv.28212 PMC9874878 36224449

[61] Más V, Melero JA. 2013. Entry of enveloped viruses into host cells: membrane fusionp 467–487. In Mateu MG (ed), Subcell Biochem. Vol. 68. 10.1007/978-94-007-6552-8_16 PMC7121288 23737062

[62] Rubiano ME, Maillard J ‐Y., Rubino JR, Ijaz MK. 2020. Use of a small‐scale, portable test chamber for determining the bactericidal efficacy of aerosolized glycol formulations. Lett Appl Microbiol 70:356–364. doi:10.1111/lam.13289 32092165

[63] Zargar B, Ijaz MK, Kevek A, Miller M, McKinney J, Sattar SA. 2024. The determination of rapid and effective activity of an air sanitizer against aerosolized bacteria using a room-sized aerobiology chamber. Microorganisms 12:2072. doi:10.3390/microorganisms12102072 39458382 PMC11510681

[64] Skowron K, Bauza-Kaszewska J, Grudlewska-Buda K, Wiktorczyk-Kapischke N, Zacharski M, Bernaciak Z, Gospodarek-Komkowska E. 2021. Nipah virus – another threat from the world of zoonotic viruses. Front Microbiol 12:811157. doi:10.3389/fmicb.2021.811157 35145498 PMC8821941

