# Supplementary material for: Rapid virucidal activity of an air sanitizer against aerosolized MS2 and Phi6 phage surrogates for non-enveloped and enveloped vertebrate viruses, including SARS-CoV-2
Source: Appl Environ Microbiol. 2024 Dec 6;91(1):e01426-24. doi: 10.1128/aem.01426-24 (PMC11784435; doi:10.1128/aem.01426-24)
Supplement: Supplemental material — Methods and results for neutralizing ability. [file aem.01426-24-s0001.pdf]

Supplemental Materials

Rapid virucidal activity of an air sanitizer against aerosolized MS2 and Phi6 phage surrogates for non-enveloped and enveloped vertebrate viruses, including SARS-CoV-2

M. Khalid Ijaz, Bahram Zargar, Raymond W. Nims, Julie McKinney & Syed A. Sattar

Method for evaluation of neutralizing ability.

Recovery agar medium (LBM double-layer agar) plates containing host *E. coli* were used for air sampling. Certain components of the LBM agar (lecithin and Polysorbate 80) were expected to immediately arrest any virucidal and/or virostatic activity in the air samples collected. To evaluate the adequacy of the chemical neutralizers used in the LBM agar, the test sanitizer was introduced into the chamber for 45 s. A 10-min air sample per plate was collected using an STA sampler containing a plate with the LBM double-layer agar. This, together withe 45-s sanitizer spray time, provided a worst-case exposure to test sanitizer for assessing neutralizing activity. Within 30 min after collection of the air sample, 100 µL of 10^{-8} dilution of the test bacteriophage suspension was spread over an LBM double-layer agar plate.

For controls, the same procedure was used to inoculate two unexposed plates containing the neutralizer, and one agar plate from the same lot was also incubated as sterility control. The plates were incubated at an appropriate temperature and observed after 18 ± 2 h of incubation. The *E. coli* plaque-forming units (PFU) on each plate were counted and recorded.

Results for evaluation of neutralizing ability.

The ability of the chemical neutralizers in the LBM agar to quench MS2 inactivation for the two test air sanitizer batches was assessed as part of the overall experimental design for this aerobiology study. The acceptance criteria for the neutralization study included: 1) the difference between the PFU counts for the two replicate control (unexposed to air sanitizer) plates (C1 and C2) must be ≤50%; and 2) the sterility check plate not exposed to MS2 must not show plaques. The acceptance criterion for adequate neutralizing activity was that the number of PFU on the agar plate exposed to test air sanitizer (NT) must be within 50% of the mean number for plates not exposed to air sanitizer (C1, C2). The assessment was performed separately for each air sanitizer batch. The results for batch 2 (Table S1) demonstrate that the neutralized condition (NT) yielded a PFU count within 24% of the mean count for the unexposed condition (C). For

batch 3 (Table S2), the neutralized condition (NT) yielded a PFU count within 23% of the mean count for the unexposed condition (C)

Table S1. Assessment of neutralizing ability for air sanitizer batch 2

Sample ID	Control(C) (Non-exposed)		Neutralizer (NT) (Exposed)	Sterility Check
**Test Date Using 45 Second Spray**: April/05/22				
**Replicate No.**	1	2	1	1
**No. of PFU**	16	25	16	0
**Average**	21		16	0
**Index=(1-NT/C) ×100%**	**(C1-C2)/Average (C1, C2) ×100%** =43%		**(1-NT/C) ×100%** =24%	No growth was observed
**Neutralization Performance:**	**Met the Criteria**		**Neutralized**	**Passed**

Table S2. Assessment of neutralizing ability for air sanitizer batch 3

Sample ID	Control(C) (Non-exposed)		Neutralizer (NT) (Exposed)	Sterility Check
**Test Date Using 45 Second Spray**: April/06/22				
**Replicate No.**	1	2	1	1
**No. of PFU**	14	12	10	0
**Average**	13		10	0
**Index=(1-NT/C) ×100%**	**(C1-C2)/Average (C1, C2) ×100%** =15%		**(1-NT/C) ×100%** =23%	No growth was observed
**Neutralization Performance:**	**Met the Criteria**		**Neutralized**	**Passed**
